# The Impact of Cardiac Magnetic Resonance Imaging on Revascularization in Ischemic Left Ventricular Dysfunction

**DOI:** 10.3390/life16060948

**Published:** 2026-06-03

**Authors:** Ghada Shalaby, Abeer Bakhsh, Aseel Jamal, Wesam Alaidarous, Ibrahim Alyazidi, Zyaad Qadi, Alaa Shafei, Sumayyah Altariqi, Ahmad Alattar, Fawaz Khateb, Ahmed Alkashghari, Yosra Turkistani, Sara Alharbi, Ahmad Samman, Fatma Aboul-Enein

**Affiliations:** 1 Department of Cardiology, King Abdullah Medical City, Makkah 21955, Saudi Arabia; 2 Cardiology Department, Faculty of Medicine, Zagazig University, Zagazig city 44519, Al Sharqia Governorate, Egypt; 3 Department of Medicine, College of Medicine, Umm Al-Qura University, Makkah 24381, Saudi Arabiafawazrk@outlook.com (F.K.);; 4 Advanced Nurse Practitioner, King Abdullah Medical City, Makkah 21955, Saudi Arabiaaltariqi.s@kamc.med.sa (S.A.); 5 Cardiology Department, Faculty of Medicine, Alexandria University, Alexandria 21526, Egypt

**Keywords:** heart failure, ischemia, microvascular obstruction, revascularization

## Abstract

Background: There are ongoing challenges in revascularizing patients with severe left ventricular (LV) dysfunction, with high rates of short- and long-term complications. Cardiac magnetic resonance (CMR) imaging is widely used for these patients; however, the prognostic value of the sum scar score and microvascular obstruction (MVO) remains unclear. Method: A retrospective study of data from King Abdullah Medical City (KAMC) between 2023 and 2025 was conducted to evaluate revascularization decisions made based on CMR studies. Patients who had normal LV function, no angiogram report, or incomplete CMR imaging were excluded. The primary outcome was hospitalization or death in patients with or without revascularization. Results: The cohort included 145 patients. The patients had a mean age of 56.7 ± 9.7 years and were predominantly male 131 (90.3%), with a high prevalence of cardiovascular risk factors: diabetes 102 (70.8%), hypertension 84 (58.7%), known dyslipidemia 46 (32%), and smoking 65 (45.5%). Most patients underwent a viability study within 7 days of a myocardial infarction (MI), 58 (40%), and a further 42 (28.9%) did so within 7–15 days. The CMR LVEF was 29.5 ± 8, and the sum scar score was 38.8 ± 21.9; MVO was observed in 34 patients (23.4%). The number of patients who underwent revascularization at the index admission was 66 (45.5%), of whom 48 (33%) received percutaneous intervention (PCI) and 18 (12.4%) underwent coronary artery bypass surgery (CABG). The patients lost to follow-up numbered 51 (35%). There were no significant differences between the two groups for the primary endpoints, including hospitalization (*p*-value: 0.61) and mortality (*p*-value: 0.31). Conclusion: In this retrospective study, CMR did not have an independent effect on hospitalization or mortality in patients who had revascularization compared to medical therapy groups. MVO and the LGE scar score were not significantly associated with cardiovascular outcomes. Our cohort was underpowered, with a high proportion of patients lost to follow-up, limiting the generalizability of the data.

## 1. Introduction

Coronary artery disease (CAD) is the most common cause of heart failure (HF) [1]. The increased prevalence of diabetes, hypertension, obesity, and dyslipidemia is associated with higher incidences of CAD and myocardial infarction (MI) [1]. The evidence supports that guideline-directed medical therapy (GDMT) improves cardiovascular outcomes; however, treatment of the primary etiology remains crucial [1]. In patients with ischemic left ventricular (LV) dysfunction, the guidelines recommend a multidisciplinary team decision for intervention and revascularization [2]. Coronary intervention is associated with a high risk of complications in patients with severe LV dysfunction [2]. The HF guidelines recommend revascularization and GDMT in patients with a low left ventricular ejection fraction (LVEF) < 35% due to CAD to improve symptoms and reduce hospitalization from cardiovascular causes and long-term mortality [1]. This recommendation is based on the long-term outcomes of the Surgical Treatment for Ischemic Heart Failure (STICH) trial [3]. However, the selection of patients who would benefit from the intervention remains controversial.

Non-invasive testing is recommended to detect ischemia-driven worsening HF, either by stress echocardiography (ECHO), positron emission tomography (PET), or cardiac magnetic resonance (CMR) imaging [1,2]. CMR imaging has high spatial resolution, aids in further characterizing the myocardium, and adds information on the LV function and volume [4,5]. Descriptive assessment of LV wall motion abnormality in the respective territory is graded as normal function, hypokinesia, or akinesia. Quantitative assessment is defined by the degree of late gadolinium enhancement (LGE), quantified as no LGE = 0, less than < 50% transmural myocardial enhancement = 1, or more than > 50% = 2 [6,7]. The LGE transmural infarction is marked for each of 17 segments, and then a sum scar score is calculated [8]. A previous paper described a correlation between scar burden and LV remodeling and improvement in LV function on follow-up, indicating the prognostic value of the LGE scar [6]. In addition, microvascular obstruction (MVO) denotes areas of acute blood-flow interruption that may correspond to hibernating myocardium [9,10]. The prognosis of these markers remains under investigation. Beyond the binary imaging assessment of myocardial viability as viable or non-viable, recent studies have proposed the extent of scar burden as a useful non-invasive prognostic factor in patients with HF [11,12]. In addition to qualitatively assessing viability by coronary territory, CMR imaging can semi-quantitatively assess scar burden using the 3-point scoring system of the 17-segment model [6,8,13]. Our previous study showed that the scar burden and viability of the left anterior descending artery territory strongly influence improvement in LV function [6,14].

There is an ongoing clinical debate regarding revascularization guided by viability. There are two major trials, STICH and the Revascularization for Ischemic Ventricular Dysfunction–British Cardiovascular Intervention Society 2 trial (REVIVED-BCIS2), which have addressed revascularization in ischemic LV dysfunction [15,16]. The Surgical Treatment for Ischemic Heart Failure (STICH) trial showed reductions in all-cause mortality and cardiovascular hospitalization (68% and 58%, respectively) that were lower in the coronary artery bypass graft surgery (CABG) arm, irrespective of viability [1,17]. The STICH trial, with an extended follow-up of 10 years, showed a favorable outcome for CABG in reducing HF-related hospitalizations [3,18]. In the International Study of Comparative Health Effectiveness with Medical and Invasive Approaches (ISCHEMIA) trial, the sub-analysis of LVEF < 45% showed that the invasive treatment arm had a lower rate of cardiovascular death and myocardial infarction (MI) at 14.6% and 25.9%, respectively [15]. The REVIVED-BCIS2 trial showed that revascularization did not provide a benefit over GDMT in terms of reducing the primary endpoint of death or hospitalization (38.2% vs. 37%) [15,16]. Anatomical and viability-guided revascularization did not affect the outcome in the REVIVED-BCIS2 study population [19].

CMR viability studies remain widely used in this population, and the prognostic value of parameters such as the sum scar score and MVO has not been well demonstrated. This study reflects real-world CMR findings in ischemic LV dysfunction and their prognostic impact.

### Objective

This study aimed to evaluate the association between CMR-driven LGE scar burden and microvascular obstruction, and their impact on revascularization strategies and clinical outcomes, in patients with ischemic LV dysfunction.

## 2. Material and Methods

### 2.1. Study Design

This is a non-randomized, retrospective, single-center study of patients who had CMR imaging for viability at King Abdullah Medical City (KAMC) from 2023 to 2025. Approval from the institutional review board (IRB) committee was obtained, and informed consent for data collection was waived.

### 2.2. Patient Selection

The inclusion and exclusion criteria for patient selection were as follows:

Inclusion criteria:

1. Patients who had a diagnosis of ischemic heart disease (IHD) or myocardial infarction (MI)-related HF.
2. Patients who had ischemic LV dysfunction with an initial LVEF by ECHO ≤ 40%.
3. HF patients who had CMR imaging for viability assessment.

Exclusion criteria:

1. Patients who had a non-ischemic cardiomyopathy.
2. Lack of baseline coronary angiogram reports.
3. Patients with preserved LV function with LVEF > 40% on ECHO.
4. Patients who had an incomplete CMR study.

### 2.3. The CMR Study

The CMR study followed the institutional protocol for viability, including post-gadolinium injection images. The study was considered incomplete and excluded if it was aborted due to claustrophobia, excessive motion, or failure to inject contrast. This was a retrospective study; no interobserver variability was performed. All the CMR studies had quantified values for LV systolic and diastolic volumes, LVEF, the right ventricle (RV) volume, and RVEF. The qualitative parameter was wall motion abnormality. The LGE was reported as transmural infarction > 50% or <50% for each myocardial wall (anterior, inferior, anterolateral, inferolateral, and septal), with correspondence to the coronary territory. The transmural sum scar burden of 17 segments was calculated for each study. MVO, as defined by a hypo-enhanced area surrounded by a hyper-enhanced scar, was reported if present in each study.

### 2.4. Study Groups

The patients who underwent percutaneous coronary intervention (PCI) or CABG after CMR were classified as the revascularized group. The decision to intervene was made in the “Heart Team” multidisciplinary team meeting, based on the clinical presentation, coronary anatomy, and the viability assessment on the CMR images.

### 2.5. Outcome

The primary outcomes are the rates of mortality and hospitalization after revascularization with either PCI or CABG compared to patients with medical therapy.

The secondary outcomes are the relationships between the sum score of the scar and microvascular obstruction, and between mortality and hospitalization.

### 2.6. Statistical Analysis

The IBM SPSS software version 26 was used for data analysis. Continuous and categorical data from the two groups were compared using a *t*-test and a chi-squared test, respectively, and Kaplan–Meier curves were generated for mortality and hospitalization. Multivariable adjusted analysis and association coefficient analysis were performed against the primary outcome. A *p*-value < 0.05 was considered significant. The patients were divided into revascularized and non-revascularized groups according to treatment strategy. The study compared the two groups with respect to demographics (e.g., age, gender, diabetes) and medication use (e.g., beta-blockers, ARNI, ACEI), echocardiography (LVEF determined by the Simpson method), CMR imaging, angiographic data, and outcomes (e.g., hospitalization, death).

## 3. Results

Patients who underwent CMR imaging between 2023 and 2024 were included in this retrospective analysis. A total of 228 patients were screened; patients who had a non-ischemic cardiomyopathy, no baseline coronary angiogram reports, left ventricular ejection fraction > 40% on echocardiography, or an incomplete CMR study were excluded. The final cohort included 145 patients. The patients were divided into revascularized and non-revascularized groups according to treatment strategy.

The mean age of the patients was 56.7 ± 9.7 years; they were predominantly male (131; 90.3%), and they had a high prevalence of cardiovascular risk factors: diabetes (102; 70.8%), hypertension (84; 58.7%), known dyslipidemia (46; 32%), and smoking (65; 45.5%). A total of 58 patients (40%) underwent a viability study within 7 days of an acute MI, and 42 (28.9%) within 7–15 days. The coronary disease involved the left anterior descending artery (LAD) in 108 (78.8%) patients and the left main (LM) artery in 12 (8.7%).

The cohort was divided by revascularization status at admission. The decision to intervene was made by the “Heart Team” in a multidisciplinary discussion, based on the patient’s age, coronary anatomy, LVEF, and CMR viability results. A total of 66 (45.5%) patients underwent revascularization, either PCI or CABG, while 79 (54.5%) had no revascularization. Of the patients who underwent revascularization, 48 (33%) had percutaneous coronary intervention (PCI) performed, and 18 (12.4%) had CABG. All patients were treated with GDMT for HF and antiplatelet therapy for acute coronary syndrome, as shown in Table 1.

The revascularized group contained more males (63; 95.4%) than the medical therapy group (68; 86%), with a *p*-value of 0.087. There were no significant differences between the two groups regarding other risk factors, including age, diabetes, hypertension, and smoking. The revascularized group had a higher proportion of patients presenting to the hospital between 15 and 30 days after the MI (19; 28.7%) than the medical therapy group (7; 8.8%), with a *p*-value = 0.004.

**Figure 1:**
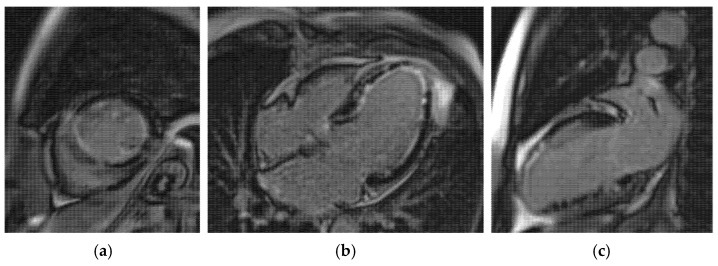
Late gadolinium enhancement (LGE) cardiovascular magnetic resonance (CMR) image in short-axis (**a**), four-chamber (**b**), and two-chamber (**c**) views demonstrating transmural myocardial infarction involving the combined left anterior descending (LAD) and right coronary artery (RCA) territories. There is extensive hyperenhancement (bright signal) indicative of irreversible myocardial injury affecting the anterior wall, interventricular septum, and inferior wall, with an area of hypoenhancement (dark, low signal) consistent with microvascular obstruction (MVO).

**Figure 2:**
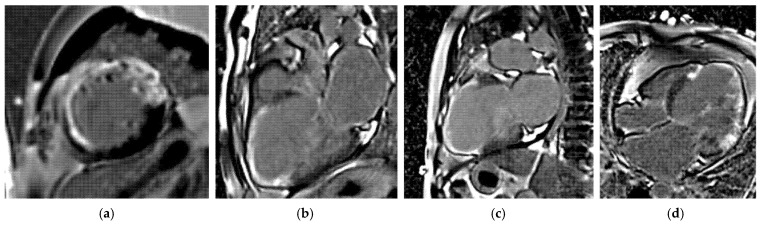
Late gadolinium enhancement (LGE) cardiovascular magnetic resonance (CMR) image in short-axis (**a**), three-chamber (**b**), two-chamber (**c**), and four-chamber (**d**) view demonstrating transmural myocardial infarction involving the combined left anterior descending (LAD) and left circumflex artery (LCX) territories. There is extensive hyperenhancement (bright signal) indicative of irreversible myocardial injury affecting the anterior wall, septum, and anterolateral walls.

**Figure 3:**
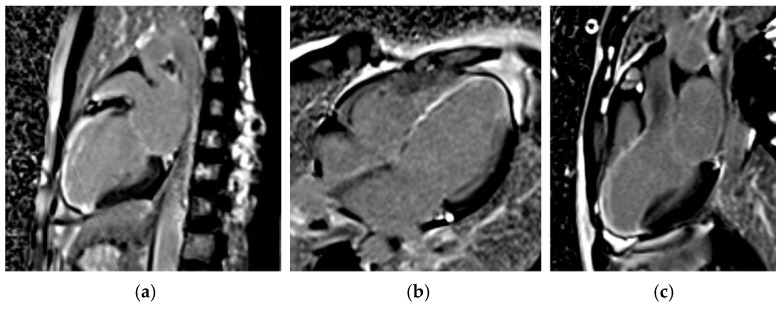
Late gadolinium enhancement (LGE) cardiovascular magnetic resonance (CMR) image in two-chamber (**a**), four-chamber (**b**), and three-chamber (**c**) view demonstrating transmural myocardial infarction involving the left anterior descending (LAD) territory. There is extensive hyperenhancement (bright signal) indicative of irreversible myocardial injury affecting the anterior wall and septal walls.

**Figure 4:**
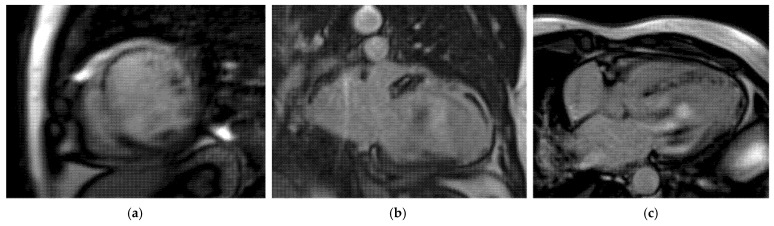
Late gadolinium enhancement (LGE) cardiovascular magnetic resonance (CMR) image in short-axis (**a**), two-chamber (**b**), and three-chamber (**c**) views demonstrating transmural myocardial infarction involving the left anterior descending (LAD) artery territory. There is extensive hyperenhancement (bright signal) indicative of irreversible myocardial injury affecting the anterior wall and interventricular septum, wall, with areas of hypoenhancement (dark, low signal) consistent with microvascular obstruction (MVO).

The ECHO showed that LV function was severely reduced, with a mean LVEF of 27 ± 8%, and LV dimensions were enlarged, with an LV end-systolic dimension (LESD) of 44.8 ± 8 mm and an LV end-diastolic dimension (LVEDD) of 55.4 ± 9 mm. RV function was preserved on average, with a tricuspid annular plane systolic excursion (TAPSE) of 17.6 ± 3 mm. Mitral regurgitation (MR) was highly prevalent, with severe MR in 13 (9.8%) cases. From CMR images, the mean LVEF was 29.5 ± 8%, with a high scar burden of LGE, with a sum scar score of 38.8 ± 21.9; and MVO was observed in 34 (23.4%) cases. The cardiac image findings are described in Table 2. The CMR findings are explained in Figure 1, Figure 2, Figure 3 and Figure 4.

There was no significant difference in LV parameters between the two groups. However, right ventricular (RV) dilatation and dysfunction were higher in the non-revascularized patients, who tended to have a larger RV size on ECHO and larger RVEDV on CMR. There was a significantly higher incidence of MVO in the medical therapy group, 24 (30%) compared to the revascularized group 10 (15.6%), with a *p*-value of 0.04. The LGE scar showed no significant difference between the two groups. The LGE scar in the medical therapy group was 39.9 ± 22.8, compared to 37.4 ± 20.8 in the revascularized group, with a *p*-value of 0.42.

**Figure 5:**
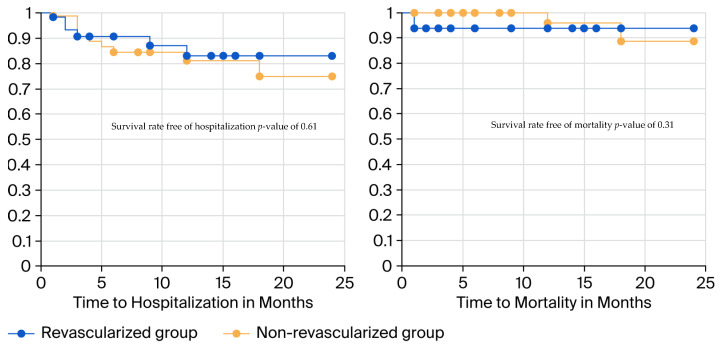
Kaplan–Meier curves for hospitalization and mortality.

The number of patients who did not receive follow-up was 51 (35%), which is related to the unique character of our center and its location in the holy capital, where pilgrims come each year for Hajj and then return to their countries afterward. Across the whole cohort, patients with active follow-up for more than a year had a mean survival of 18 months, and the hospitalization rate at 12 months was 4.3%. In patients who had revascularization, the survival rate free of hospitalization was 98% at 1 month and 83% by 12 months. The patients on medical therapy had a survival rate free of hospitalization of 98.7% at 1 month and 96% at 12 months. There was no significant difference in the rate of hospitalization between the two groups, with a *p*-value of 0.61. In patients who received revascularization, the survival rate was 93.8% at 1 month, and no further deaths occurred by 12 months. The mortality occurred late in the medical therapy group; the survival rate at 12 months was 96%, yielding a non-significant *p*-value: 0.31 (Figure 5). Hospitalization for cardiovascular causes occurred for 16 (11%) patients overall: six (9%) in the revascularized group and 10 (12.6%) in the medical therapy group, with a *p*-value of 0.67. Mortality occurred in six (4%) patients: four (6%) in the revascularized group and two (2.5%) in the medical therapy group, with a *p*-value of 0.5, as shown in Table 3. There were no significant differences between the two groups in the primary endpoints, including hospitalization and mortality; however, hospitalization was higher among non-revascularized patients, and mortality was higher among patients with attempted intervention. In the revascularized group, four mortalities occurred within 30 days, due to cardiogenic shock, ventricular arrhythmia, and post-procedure bleeding (one patient post-PCI and one patient post-CABG). The other two patients died due to advanced HF in the medical therapy group after 1 year.

For the overall cohort, there was no significant association between comorbidity and mortality or hospitalization. The coronary anatomy was associated with increased mortality, with involvement of the left anterior descending (LAD) artery (*p*-value: 0.029). Mortality in the revascularization group showed no significant association with MI timing, with a mortality *p*-value of 0.29 and a hospitalization *p*-value of 0.17. Mortality in the medical therapy group showed a slightly higher association with MI timing, with a mortality *p*-value of 0.07, while showing no significant association with hospitalization (*p*-value: 0.2).

Multivariable adjusted analysis was performed on the prespecified eight predictors based on the clinical rationale of clinical risk factors, LVEF, and CMR scar burden, to minimize overfitting (Table 4). The analysis was performed against a composite of hospitalization and mortality. The multivariable analysis should be interpreted with caution, given the inverse relationship between LVEF and scar burden, the small sample size, and model overfitting. Female gender showed a favorable odds ratio. However, the analysis has limitations, including a low events-per-variable ratio and a single-center retrospective design. In addition, the CMR was performed early post-MI, leading to an overestimation of scar burden.

Patients with MVO had a mean LVEF of 29.2 ± 8.8%, which was not statistically different from those without MVO. The sum scar score of 50 ± 19.8 was significantly higher in patients with MVO than those without MVO (34.9 ± 20), with a *p*-value of 0.001. The CMR findings in patients with MVO are described in Table 5.

Of the patients who had revascularization at the time of admission, 10 (29.4%) had MVO, compared to 55 (50.5%) without MVO, with a *p*-value of 0.037. Hospitalization for cardiac causes occurred in four (11.7%) patients with MVO; in contrast, patients without MVO had more hospitalizations, 12 (11%), but this difference was not statistically significant, as shown in Table 6. When adjusted for comorbidity, the presence of MVO was higher when associated with hypertension (*p*-value: 0.038) and smoking (*p*-value: 0.045). There was no significant relation with age (*p*-value: 0.18) or diabetes (*p*-value: 0.27). When adjusted for coronary anatomy, the left anterior descending artery was not associated with higher MVO; however, the left circumflex artery was associated with a higher prevalence of MVO. The timing of MI was not significantly associated with the presence of MVO.

The number of patients with an LGE sum scar score >50%, denoting extensive scarring, was 48 (33.6%), with a mean scar score of 62 ± 10.8. The number of patients who had revascularization at the time of admission was 16 (33%), of whom 13 (27%) had PCI and three (6%) had CABG. In contrast, the number of patients with an LGE sum scar score < 50% was 95 (66.4%), with a mean scar score of 26.7 ± 14. The number of patients who had revascularization at the time of admission was 49 (51.6%), of whom 35 (36.8%) had PCI and 14 (14.7%) had CABG. The patients with an LGE sum scar score >50% had hospitalization due to cardiac causes in seven (14.6%) cases. Patients with lower scar burden had similar hospitalization rates (9; 9.5%), with a *p*-value of 0.057 (Table 7).

**Figure 6:**
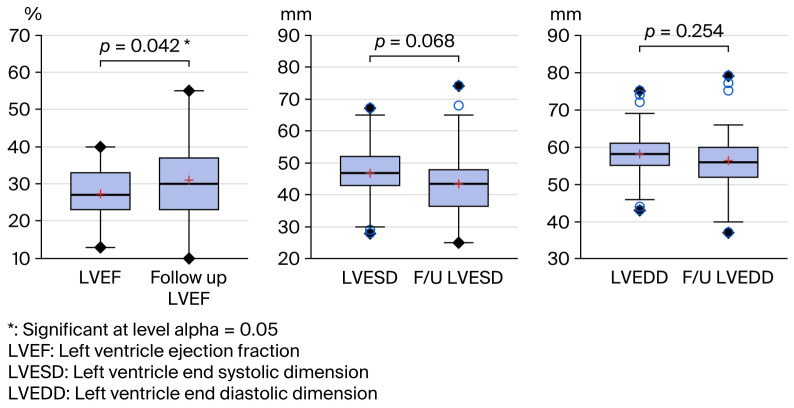
Follow-up echocardiographic parameters for the whole cohort are shown in boxplot.

The number of patients who had follow-up ECHO was 53 (36.6%). For these patients, there was a significant relationship between baseline and follow-up LVEF, with a mean LVEF at baseline of 27 ± 8%, and at follow-up of 31 ± 11%, with a *p*-value of 0.042 (Figure 6). The mean LVESD at baseline was 46.8 ± 8, and on follow-up was 43.5 ± 11.5 mm, with a *p*-value of 0.068, while the LVEDD at baseline was 58 ± 7, and on follow-up was 56 ± 8 mm, with a *p*-value of 0.254. There was an improvement in LVEF on follow-up in both groups. For patients who underwent revascularization, the LVEF changed from 28.5 ± 7.5% at baseline to 32.5 ± 11% on follow-up, with a *p*-value of 0.16. For the medical therapy group, LVEF increased from 25.5 (8.5%) at baseline to 29 (11%) on follow-up, with a *p*-value of 0.18. There was a relative improvement in the LVEF and the LVESD over the follow-up period, irrespective of the intervention.

## 4. Discussion

The utility of viability studies as a decision-making tool for patient treatment strategy remains controversial. The current retrospective study included 145 patients with ischemic left ventricular dysfunction, of whom 66 (45.5%) underwent revascularization, either by PCI or CABG, based on CMR viability study results. The results showed no significant differences between the two groups regarding the primary endpoints, including hospitalization and mortality. However, mortality was higher among the revascularized group, given that four patients died due to cardiogenic shock, ventricular arrhythmia, and periprocedural bleeding. The guidelines on revascularization for CAD with LV dysfunction (LVEF ≤ 35%) recommend that, after evaluation and discussion in the Heart Team meeting, revascularization be performed based on coronary anatomy, comorbidities, the relationship between CAD and HF, and patient preference [2]. These guidelines were largely informed by evidence from large clinical trials, such as STICH, ISCHEMIA, and REVIVED-BCIS2 [2,15]. These trials did not show any benefit of revascularization over GDMT in ischemic LV dysfunction [18].

These data pertain to stable or chronic CAD, whereas in the real-world data, many patients had a viability evaluation soon after an MI. Most patients had a viability study within 7 days (58; 40%), or within 7–15 days (42; 28.9%). However, the timing of MI was similar in both treatment arms. The viability in our study was quantified using the LGE scar score, which was similar in both groups; however, patients with an LGE scar burden < 50% (viable) tended to undergo more revascularization. The number of patients with an LGE sum scar score < 50% was 95 (66.4%), of whom 49 (51.6%) underwent intervention: 35 (36.8%) PCI and 14 (14.7%) CABG. The REVIVED-BCIS2 trial showed that PCI plus GDMT, compared with GDMT alone, failed to improve LV function, heart failure outcomes, or survival, even in patients with a viable myocardium [16].

MVO was observed in 34 patients (23.7%) in this cohort. There was a significantly higher incidence of MVO in the medical therapy group (24; 30%) compared to the revascularized group (10; 15.6%), with a *p*-value of 0.04. The presence of MVO was not significantly associated with MI timing. The mean LVEF was similar in patients with and without MVO; the LGE scar score was higher in the MVO group. The presence of MVO did not contribute to the long-term outcome. In a retrospective analysis of 597 patients post-MI, MVO was a significant predictor of all-cause mortality, especially in patients with LVEF > 35% [10].

### 4.1. Limitations

This study has many limitations, mainly due to its retrospective design, which is subject to selection bias. The study is underpowered for survival outcomes, and a large number of patients were lost to follow-up after the initial admission, which limits the ability to draw definite conclusions on mortality and hospitalization.

### 4.2. Clinical Implication

The CMR for viability did have a significant association with cardiovascular-related hospitalization or mortality. The role of optimal GDMT remains pivotal in managing patients with ischemic LV dysfunction, with or without intervention. Coronary revascularization can be personalized through a multidisciplinary team approach.

## 5. Conclusions

The current study did not demonstrate a relationship between viability and clinical outcomes. Given the study’s limitation due to its retrospective design, underpowered state, and large number of patients lost to follow-up, these findings cannot be generalized. Large prospective studies are needed to correlate the LGE scar score and MVO with clinical outcomes and LV remodeling.

## Figures and Tables

**Table 1:** Baseline characteristics of patients with ischemic left ventricular dysfunction.

	All N = 145	Non-Revascularized N = 79 (54.5%)	Revascularized N = 66 (45.5%)	*p*-Value
Demographics				
Age (years)	56.7 ± 9.7	56 ± 10	57 ± 9	0.44
Gender				
Male	131 (90%)	68 (86%)	63 (95.4%)	0.087
Female	14 (9.6%)	11 (13.9%)	3 (4.5%)	0.087
Comorbidity				
Diabetes	102 (70.8%)	56 (70.8%)	46 (70.7%)	1
Hypertension	84 (58.7%)	44 (56.4%)	40 (61.5%)	0.66
Dyslipidemia	46 (32%)	25 (32%)	21 (32%)	1
Smoking	65 (45.5%)	34 (43.6%)	31 (47.6%)	0.75
Onset of myocardial infarction				
<7 days	58 (40%)	36 (45.5%)	22 (33%)	0.17
7–15 days	42 (28.9%)	23 (29%)	19 (28.7%)	1
15–30 days	26 (17.9%)	7 (8.8%)	19 (28.7%)	0.004
>30 days	11 (7.5%)	5 (6.3%)	6 (9%)	0.75
Undefined	8 (5.5%)	8 (10%)	0	0.01
Coronary anatomy				
Left main	12 (8.7%)	4 (5.6%)	1 (8%)	0.45
Left anterior descending	108 (78.8%)	48 (67.6%)	60 (90.9%)	<0.001
Left circumflex	61 (44.5%)	22 (30.9%)	39 (59%)	0.001
Right coronary	68 (49.6%)	27 (38%)	41 (62%)	0.001
Pharmacotherapy				
Beta-blockers				
Metoprolol	1 (0.7%)			
Bisoprolol	133 (95%)	73 (94.8%)	60 (95%)	0.98
Carvedilol	2 (1.4%)	1 (1.3%)	1 (1.5%)	1
Angiotensin-converting enzyme inhibitor				
Perindopril	13 (9.2%)	5 (6.5%)	8 (12.7%)	0.36
Lisinopril	12 (8.5%)	6 (7.8%)	6 (9.5%)	0.98
Angiotensin receptor blockers				
Valsartan	50 (35.7%)	26 (33.7%)	24 (38%)	0.79
Irbesartan	1 (0.7%)			
ARNI				
Dose: 50 mg	40 (28.5%)	22 (28.5%)	18 (28.5%)	1
Dose: 100 mg	21 (15%)	14 (18%)	7 (11%)	0.3
Dose: 200 mg	10 (7%)	7 (9%)	3 (4.7%)	0.47
Loop diuretics	112 (80%)	60 (77.9%)	52 (82.5%)	0.8
Mineralocorticoids	129 (92%)	71 (92%)	58 (92%)	0.9
Antiplatelets				
Clopidogrel	106 (75.7%)	54 (70%)	52 (82.5%)	0.04
Ticagrelor	13 (9%)	4 (5%)	9 (14%)	0.14
ASA	120 (85.7%)	61 (79%)	59 (93.6%)	0.075
Anticoagulant				
Warfarin	9 (6%)	4 (5.2%)	5 (7.9%)	0.78
Apixaban	28 (20%)	23 (29.8%)	5 (7.9%)	0.001
Vasodilator				
Hydralazine	6 (4%)	4 (5.2%)	2 (3%)	0.8
Isosorbide	40 (28.5%)	22 (28.6%)	18 (28.5%)	1
Statin therapy				
Atorvastatin	89 (63.5%)	47 (61%)	42 (66.6%)	0.73
Rosuvastatin	46 (32.8%)	26 (33.8%)	20 (31.7%)	0.87
Device				
Implantable defibrillator	7 (5%)	4 (5.3%)	3 (4.7%)	1
Cardiac resynchronization therapy	4 (2%)	3 (3.9%)	1 (1.5%)	0.7
Permanent pacemaker	1 (0.7%)			
Revascularization at the index of admission	66 (45.5%)			
Percutaneous coronary intervention	48 (33%)			
Coronary artery bypass graft surgery	18 (12.4%)			

**Table 2:** Baseline cardiac imaging parameters.

	All N = 145	Non-Revascularized N = 79 (54.5%)	Revascularized N = 66 (45.5%)	*p*-Value
Echocardiography				
LVEF (%)	27 ± 8	26 ± 8	28 ± 8	0.29
LVESD (mm)	44.8 ± 8	45.7 ± 9.8	43.6 ± 9.4	0.18
LVEDD (mm)	55.4 ± 9	56 ± 9.3	54.7 ± 8.7	0.18
RV size (mm)	25.8 ± 7.8	26.9 ± 7.9	24.4 ± 7.6	0.078
TAPSE (mm)	17.6 ± 3	17.8 ± 3.6	17.5 ± 2.7	0.84
S’ (m/s)	106.5 ± 26	103 ± 26	111 ± 25	0.14
Degree of mitral regurgitation				
Mild	65 (49%)	36 (50%)	29 (48%)	
Moderate	22 (16.6%)	9 (12.5%)	13 (21.6%)	
Severe	13 (9.8%)	8 (11%)	5 (8.3%)	
CMR				
LVEF (%)	29.5 ± 8	29.4 ± 8.5	29.7 ± 7.8	0.64
LVESV (mL)	102.6 ± 61	106 ± 67	98 ± 52.7	0.57
LVEDV (mL)	138 ± 72	142.6 ± 80	133 ± 61.6	0.65
RVEF (%)	49 ± 13	47 ± 13.6	51 ± 13	0.14
RVEDV (mL)	73.8 ± 32	79.6 ± 25	67 ± 25	0.061
Sum score of LGE scar	38.8 ± 21.9	39.9 ± 22.8	37.4 ± 20.8	0.42
Left ventricle thrombus	21 (15%)	12 (15.7%)	9 (14%)	0.97
Microvascular obstruction	34 (23.4%)	24 (30%)	10 (15.6%)	0.04

CMR: cardiac magnetic resonance imaging; LVEF: left ventricular ejection fraction; LVESD: left ventricular end-systolic dimension; LVEDD: left ventricular end diastolic dimension; RV: right ventricle; TAPSE: tricuspid annular plane systolic excursion; LVESV: left ventricular end-systolic volume; LVEDV: left ventricular end diastolic volume; RVEF: right ventricular ejection fraction; RVEDV: right ventricular end diastolic volume; LGE: late gadolinium enhancement.

**Table 3:** Outcome of the cohort after revascularization compared to no revascularization.

	All N = 145	Non-Revascularized N = 79 (54.5%)	Revascularized N = 66 (45.5%)	*p*-Value
Loss of follow-up	51 (35%)	28 (35.4%)		1
Hospitalization	16 (11%)	10 (12.6%)	6 (9%)	0.67
Mortality	6 (4%)	2 (2.5%)	4 (6%)	0.5

**Table 4:** Multivariable logistic regression for composite of hospitalization and mortality.

Variable	β (SE)	Wald	*p*-Value	Odds Ratio	95% CI
Age	−0.032 (0.032)	0.988	0.320	0.969	[0.910, 1.031]
Female sex	−1.954 (0.741)	6.954	0.008	0.142	[0.033, 0.606]
Diabetes mellitus	−0.029 (0.579)	0.002	0.960	0.971	[0.312, 3.024]
Dyslipidemia	0.086 (0.555)	0.024	0.877	1.090	[0.367, 3.235]
Hypertension	−0.738 (0.607)	1.480	0.224	0.478	[0.145, 1.570]
Smoking	−0.758 (0.621)	1.489	0.222	0.469	[0.139, 1.583]
Scar burden (sum/34)	−0.033 (0.013)	6.078	0.014	0.968	[0.943, 0.993]
CMR ejection fraction	−0.082 (0.036)	5.025	0.025	0.922	[0.858, 0.990]
Constant	5.787 (2.577)	5.042	0.025	326.109	—

Model fit: Omnibus χ^2^(8) = 16.86, *p* = 0.032; Nagelkerke R^2^ = 0.202; n = 141; events = 20.

**Table 5:** Characteristics of patients with microvascular obstruction as determined by CMR imaging.

	MVO Present N = 34 (23.4%)	MVO Absent N = 111 (76.5%)	*p*-Value
LVEF	29.2 ± 8.8	29.6 ± 7.8	0.9
LVEDV	141 ± 60	138 ± 73	0.62
LVESV	99.5 ± 47	104 ± 57	0.68
LGE sum scar score	50 ± 19.8	34.9 ± 20	0.001

CMR: cardiac magnetic resonance; MVO: microvascular obstruction; LVEF: left ventricular ejection fraction; LVESV: left ventricular end-systolic volume; LVEDV: left ventricular end-diastolic volume; LGE: late gadolinium enhancement.

**Table 6:** Outcome of the cohort with and without microvascular obstruction.

	MVO Present N = 34 (23.4%)	MVO Absent N = 111 (76.5%)	*p*-Value
Revascularization	10 (29.4%)	55 (50.5%)	0.037
Hospitalization	4 (11.7%)	12 (11%)	1
Mortality	0	6 (5.5%)	0.1

MVO: microvascular obstruction.

**Table 7:** Outcome of the cohort based on the LGE scar score.

	Sum Scar Score > 50% N = 48 (33.6%)	Sum Scar Score < 50% N = 95 (66.4%)	*p*-Value
Revascularization	16 (33%)	49 (51.5%)	0.05
Hospitalization	7 (14%)	9 (9.5%)	0.54
Mortality	0	6 (6%)	0.057

LGE: late gadolinium enhancement.

## Data Availability

Data are contained within the article.

## Abbreviations

ACS	Acute coronary syndrome
CAD	Coronary artery disease
CMR	Cardiac MRI
DM	Diabetes mellitus
EF	Ejection fraction
GDMT	Guideline-directed medical therapy
HTN	Hypertension
IHD	Ischemic heart disease
LM	Left main
LV	Left ventricular
MI	Myocardial infarction
MVO	Microvascular obstruction
NSTEMI	Non-ST elevation myocardial infarction
OMT	Optimal medical therapy
PCI	Percutaneous coronary intervention

## References

[1] Heidenreich P.A., Bozkurt B., Aguilar D., Allen L.A., Byun J.J., Colvin M.M., Deswal A., Drazner M.H., Dunlay S.M., Evers L.R. (2022). 2022 AHA/ACC/HFSA Guideline for the Management of Heart Failure: Executive Summary: A Report of the American College of Cardiology/American Heart Association Joint Committee on Clinical Practice Guidelines. J. Am. Coll. Cardiol..

[2] Vrints C., Andreotti F., Koskinas K.C., Rossello X., Adamo M., Ainslie J., Banning A.P., Budaj A., Buechel R.R., Chiariello G.A. (2024). 2024 ESC Guidelines for the management of chronic coronary syndromes: Developed by the task force for the management of chronic coronary syndromes of the European Society of Cardiology (ESC) Endorsed by the European Association for Cardio-Thoracic Surgery (EACTS). Eur. Heart J..

[3] Matthew Ryan D.P. (2023). CHRONIC HEART FAILURE Revascularization strategies in patients with low left ventricular function. The PCR-EAPCI Textbook.

[4] Kabakus I.M., Chamberlin J.H., Miller E.J. (2024). Cardiac MRI assessment of myocardial viability in chronic myocardial infarction: How should we do it?. Front. Cardiovasc. Med..

[5] Rizzello V., Poldermans D., Boersma E., Biagini E., Schinkel A.F., Krenning B., Elhendy A., Vourvouri E.C., Sozzi F.B., Maat A. (2004). Opposite patterns of left ventricular remodeling after coronary revascularization in patients with ischemic cardiomyopathy: Role of myocardial viability. Circulation.

[6] Aboul Enein F., Allaaboun S., Khayyat S., Andijani M., Alkhuzai M.M., Aljunied A.A., Al Adhreai M.S. (2020). Association Between Myocardial Scar Burden and Left Ventricular Ejection Fraction in Ischemic Cardiomyopathy. Cureus.

[7] Al-Sabeq B., Nabi F., Shah D.J. (2019). Assessment of myocardial viability by cardiac MRI. Curr. Opin. Cardiol..

[8] Segmentation A.H.A.W.G.o.M., Imaging R.f.C., Cerqueira M.D., Weissman N.J., Dilsizian V., Jacobs A.K., Kaul S., Laskey W.K., Pennell D.J., Rumberger J.A. (2002). Standardized Myocardial Segmentation and Nomenclature for Tomographic Imaging of the Heart. Circulation.

[9] Nicolau A.M., Silva P.G., Mejia H.P.G., Granada J.F., Kaluza G.L., Burkhoff D., Abizaid T., Pileggi B., Freire A.F.D., Godinho R.R. (2025). Molecular Mechanisms of Microvascular Obstruction and Dysfunction in Percutaneous Coronary Interventions: From Pathophysiology to Therapeutics-A Comprehensive Review. Int. J. Mol. Sci..

[10] Brado J., Schmitt R., Hein M., Valina C., Steinhauer C., Soschynski M., Schuppert C., Schlett C.L., Neumann F.J., Westermann D. (2025). Predicting MRI-diagnosed microvascular obstruction and its long-term impact after acute myocardial infarction. Clin. Res. Cardiol..

[11] Ahmed S.W., Sultan F.A.T., Awan S., Ahmed I. (2020). Prognostic Significance of CMR Findings in Patients with Known Coronary Artery Disease—Experience from a South Asian Country. J. Clin. Imaging Sci..

[12] Prameswari H.S., Kamarullah W., Pranata R., Putra I.C.S., Undarsa A.C., Iqbal M., Dewi T.I., Kusumawardhani N.Y., Akbar M.R., Astuti A. (2025). Meta-analysis of cardiac magnetic resonance in prognosticating left ventricular function in peripartum cardiomyopathy. ESC Heart Fail..

[13] Porcari A., De Luca A., Grigoratos C., Biondi F., Faganello G., Vitrella G., Nucifora G., Aquaro G.D., Merlo M., Sinagra G. (2022). Arrhythmic risk stratification by cardiac magnetic resonance tissue characterization: Disclosing the arrhythmic substrate within the heart muscle. Heart Fail. Rev..

[14] Laymouna R., El-Sharkawy E., El-Taha S., Elfiky M. (2023). Prognostic value of myocardial scar in ischaemic and non-ischaemic cardiomyopathy using cardiac magnetic resonance imaging. Cardiovasc. J. Afr..

[15] Virani S.S., Newby L.K., Arnold S.V., Bittner V., Brewer L.C., Demeter S.H., Dixon D.L., Fearon W.F., Hess B., Johnson H.M. (2023). 2023 AHA/ACC/ACCP/ASPC/NLA/PCNA Guideline for the Management of Patients With Chronic Coronary Disease: A Report of the American Heart Association/American College of Cardiology Joint Committee on Clinical Practice Guidelines. Circulation.

[16] Perera D., Clayton T., O’Kane P.D., Greenwood J.P., Weerackody R., Ryan M., Morgan H.P., Dodd M., Evans R., Canter R. (2022). Percutaneous Revascularization for Ischemic Left Ventricular Dysfunction. N. Engl. J. Med..

[17] Ryan M., Truesdell A.G., Murphy G.J., Ezad S.M., Fremes S., Lansky A.J., Omerovic E., Windecker S., Velazquez E.J., Petrie M.C. (2025). Revascularization in Ischemic Left Ventricular Dysfunction: A Pathophysiology-Guided, Evidence-Based Approach. JACC Cardiovasc. Interv..

[18] Howlett J.G., Stebbins A., Petrie M.C., Jhund P.S., Castelvecchio S., Cherniavsky A., Sueta C.A., Roy A., Piña I.L., Wurm R. (2019). CABG Improves Outcomes in Patients With Ischemic Cardiomyopathy. JACC Heart Fail..

[19] Ezad S.M., McEntegart M., Dodd M., Didagelos M., Sidik N., Li Kam Wa M., Morgan H.P., Pavlidis A., Weerackody R., Walsh S.J. (2024). Impact of Anatomical and Viability-Guided Completeness of Revascularization on Clinical Outcomes in Ischemic Cardiomyopathy. J. Am. Coll. Cardiol..

